# Supplementary material for: Low Levels of Hive Stress Are Associated with Decreased Honey Activity and Changes to the Gut Microbiome of Resident Honey Bees
Source: Microbiol Spectr. 2023 Jun 8;11(4):e00742-23. doi: 10.1128/spectrum.00742-23 (PMC10434159; doi:10.1128/spectrum.00742-23)
Supplement: Supplemental file 1 — Supplemental material. Download spectrum.00742-23-s0001.docx, DOCX file, 0.03 MB [file spectrum.00742-23-s0001.docx]

**Supplementary Material S1**

**Honey Sample Preparation**

Unless otherwise specified, all honey samples were mixed thoroughly with a spatula, incubated at 35 °C for 30 minutes to dissolve crystals, diluted to the target concentration in sterile water, and vortexed thoroughly before use. Control honeys with known activity levels included in all assays were artificial honey (1.5 g sucrose, 7.5 g maltose, 40.5 g fructose, 33.5 g glucose, 17 mL sterile water), peroxide-activity-based Barnes TA 10+ Jarrah honey, and non-peroxide-activity-based Comvita UMF 18+ Manuka honey.

**Test Strains & Culture Conditions**

Strains used were two Gram-positive bacteria (*Staphylococcus aureus* ATCC29213*, Enterococcus faecalis* ATCC29212), two Gram-negative bacteria (*Pseudomonas aeruginosa* ATCC29883, *Escherichia coli* ATCC25923), two yeasts (*Candida dubliniensis* AMMRL1881, *Cryptococcus deuterogattii* R265), and two moulds (*Aspergillus flavus* ATCC204304*, Trichophyton interdigitale* MN608077). Bacterial strains, yeast strains, and *A. flavus* were maintained as glycerol stocks at –80 °C. Bacterial strains were grown on Nutrient Agar (NA; Oxoid) and incubated at 30 °C for 24 hours before use. Yeast strains and *A. flavus* were grown on Potato Dextrose Agar (PDA; Oxoid) and incubated at 30 °C for 24 – 48 hours. *T. interdigitale* was maintained on an agar slope, grown on Oatmeal Agar (Sigma Aldrich), and incubated at 30 °C for up to 7 days until good sporulation was obtained.

**Antimicrobial Susceptibility Testing by Broth Microdilution**

Antimicrobial susceptibility testing by broth microdilution in 96-well plates was performed in accordance with CLSI guidelines for aerobic bacteria, yeasts, and filamentous fungi with minor modifications. Briefly, inocula were prepared from colonies growing on agar plates to a final concentration of 2 x 10^5^ – 8 x 10^5^ for bacteria, 0.5 x 10^3^ – 2.5 x 10^3^ CFU/ml for yeasts, 0.4 x 10^4^ – 5 x 10^4^ for *A. flavus* and 1 x 10^3^ – 3 x 10^3^ CFU/ml for *T. interdigitale*. Bacterial strains were adjusted to an absorbance of between 0.08 – 0.1 at 625 nm while fungal strains were counted using a haemocytometer. Assays used Mueller-Hinton Broth (MHB; Oxoid) supplemented with 20 mg Ca^++^/L and 10 mg Mg^++^/L for bacteria, RPMI-1640 (Sigma-Aldrich) supplemented with 0.165 M MOPS and 2% D-glucose for *C. dubliniensis* and moulds, and Yeast Nutrient Broth (YNB; Sigma-Aldrich) supplemented with 0.165 M MOPS and 0.5% D-glucose for *C. deuterogattii*. Honeys were assayed at 5, 10, 15, 20, 25, and 30% (w/v) diluted in either sterile water (for total activity) or freshly prepared 5600 U/ml catalase solution (for non-peroxide activity). Tetracycline was included as a drug control for bacterial strains and amphotericin B as a drug control for fungal strains. Plates were incubated without agitation at 35 °C for 20 hours (bacteria), 48 hours (yeasts and *A. flavus*) or 96 hours (*T. interdigitale*). The MIC was determined visually and defined as the lowest drug concentration at which growth was inhibited 100%. Three independent biological repeats were performed for each honey sample.

**Preparation of Honey Phenolic Extracts**

In 15 mL tubes, 3 mL of acetonitrile was added to 1 mL of 50% (w/v) honey. The mixture was vortexed for 30 seconds and allowed to settle for 5 min at RT forming two layers. The acetonitrile top layer was transferred to a pre-weighed glass test tube, and an additional 5 mL of fresh acetonitrile added to the water bottom layer and the extraction process repeated. The new acetonitrile top layer was transferred to the glass test tube and mixed with the first extract. The total extract containing mainly phenolic compounds was evaporated under vacuum using a GeneVac EZ-2 Centrifuge and weighed to determine the final mass. The sample was reconstituted in methanol at 10 mg/mL and stored in the dark at -20 °C overnight before HPLC profiling analysis.

**DNA Preparation**

For gut samples, bee bodies were surface sterilised in 1% NaOCl for 3 min and rinsed in sterile water for 30 sec. Sterile forceps were used to extract the digestive tract by pulling on the last segment of the abdomen and the crop, midgut, and hindgut were carefully separated and stored at –30 °C until further processing. Gut dissections were performed for 20 bees per hive and pooled together. For pollen samples, 100 mg was measured out. For swab samples, swabs were removed from transport media and the swab heads cut off using sterile scissors, removing any hard plastic pieces. All samples were then transferred to tubes containing 500 mg of 2 mm glass beads in 500 µl of PBS and homogenised in a bead beater using 3 cycles of 30 sec at 3000 rpm with 30 sec rests between. DNA extractions were performed using DNeasy Blood & Tissue Kits (Qiagen) according to manufacturer instructions. DNA concentration was measured using the Qubit dsDNA BR Assay Kit (Invitrogen). PCR and gel electrophoresis was conducted to confirm the presence of sufficient bacterial and fungal DNA in samples, with details of primers and PCR conditions in Table S1.1.

**Amplicon Sequencing & Analysis**

DNA was sent to Ramaciotti Centre for Genomics at the University of New South Wales, Sydney for 16S rRNA gene V3-V4 amplicon sequencing with the 341F-805R primer set using the Illumina Miseq v3 2x300 bp platform, and to BGI Genomics, Hong Kong for ITS1 amplicon sequencing with the ITS1F-ITS2 primer set using the DNBSEQ PE300 platform. Raw sequence reads were processed in R v4.2.2 using the DADA2 pipeline to generate amplicon sequence variants (ASVs). Default parameters were used to filter and trim, learn error rates, merge paired reads, and remove chimeras with the following adjustments: the truncLen parameter was adjusted to c(260, 220) to allow for sufficient overlap of forward and reverse reads for merging of the V3-V4 amplicons, and this step was not performed for the variable length ITS1 amplicons. After processing, there was an average of 75005 reads per test sample, and 574 in the H_2_O negative control.Taxonomy was assigned using the SILVA database release 138.1 for 16S and the UNITE database release 27.10.2022 for ITS. Non-bacteria, mitochondria and chloroplast were filtered out from 16S taxonomic tables and non-fungi from ITS taxonomic tables. Alpha diversity, beta diversity, and taxonomic relative abundance were calculated and visualised using the phyloseq and ggplot2 R packages.

**Table S1.1. Primers and PCR conditions**

| **Primer** | **Sequence (5’ – 3’)** | **PCR Step** | **Conditions** | **Cycles** |
| --- | --- | --- | --- | --- |
| **16S V3-V4** | | | | |
| 341F  805R | CCTACGGGNGGCWGCAG  GACTACHVGGGTATCTAATCC | Initial Denaturation | 94 °C for 30 s | 1 |
|  |  | Denaturation | 94 °C for 30 s | 25 |
|  |  | Annealing | 60 °C for 30 s |  |
|  |  | Extension | 68 °C for 90 s |  |
|  |  | Final Extension | 68 °C for 5 m | 1 |
| **ITS1** | | | | |
| ITS1F  ITS2 | CTTGGTCATTTAGAGGAAGTAA  GCTGCGTTCTTCATCGATGC | Initial Denaturation | 94 °C for 30 s | 1 |
|  |  | Denaturation | 94 °C for 30 s | 25 |
|  |  | Annealing | 60 °C for 30 s |  |
|  |  | Extension | 68 °C for 90 s |  |
|  |  | Final Extension | 68 °C for 5 m | 1 |
